# Co-Occurrence of Inflammatory Bowel Disease in Patients with Vitiligo: A Systematic Review and Meta-Analysis

**DOI:** 10.5152/tjg.2026.25282

**Published:** 2026-01-02

**Authors:** Zhaoyi Gu, Jingjing Teng

**Affiliations:** 1 Department of Gastroenterology, Suzhou Science & Technology Town Hospital, Suzhou, China

**Keywords:** Inflammatory bowel diseases, meta-analysis, prevalence, vitiligo

## Abstract

**Background/Aims:**

Inflammatory bowel disease (IBD) is a chronic, increasingly common condition linked to other autoimmune disorders, including vitiligo. This study aims to assess IBD co-occurrence in vitiligo patients to explore underlying immunogenetic factors and inform better screening and management.

**Materials and Methods:**

A systematic search was conducted across Scopus, PubMed/Medline, Web of Science, and Embase, to identify relevant studies published up to March 31, 2025. Studies examining the prevalence of IBD and its subtypes in patients with vitiligo were included. Data were extracted and pooled using random effects models, with heterogeneity assessed through Cochran’s Q test and Higgins *I* ^2^ test.

**Results:**

A total of 16 studies with 44 records were included in the analysis. The pooled prevalence of IBD was 0.97% (95% CI: 0.69-1.25), with Crohn’s disease (CD) and ulcerative colitis (UC) reported at 0.45% (0.32-0.59) and 0.69% (0.42-0.97), respectively. Also, vitiligo was significantly associated with an increased risk of IBD (odds ratio [OR] = 1.51, 95% CI: 1.10-2.07), particularly for CD (OR = 1.49, 95% CI: 1.13-1.97). The pooled prevalence of IBD varied across countries, with the highest prevalence reported in Saudi Arabia. Additionally, women with vitiligo had a slightly higher pooled prevalence of IBD compared to men.

**Conclusion:**

This study found that the prevalence of IBD in patients with vitiligo is approximately 0.97%, with vitiligo significantly increasing the risk of developing IBD, particularly CD. Clinicians should consider IBD in the differential diagnosis of vitiligo patients with gastrointestinal symptoms.

**Main Points**

- The pooled prevalence of inflammatory bowel disease (IBD) in patients with vitiligo was found to be 0.97%, with Crohn’s disease (CD) and ulcerative colitis prevalences at 0.45% and 0.69%, respectively.
- Vitiligo significantly increases the risk of developing IBD, with an odds ratio (OR) of 1.51, particularly for CD (OR = 1.49).
- The highest prevalence of IBD in vitiligo patients was reported in Saudi Arabia (1.72%), while women showed a slightly higher pooled prevalence compared to men.
- No significant differences in IBD prevalence were observed across geographic regions or over time, despite variations in study methodologies and populations.
- Clinicians should consider IBD as a differential diagnosis in vitiligo patients presenting with gastrointestinal symptoms due to the identified association.

## Introduction

Inflammatory bowel disease (IBD) is a chronic and inflammatory disorder caused by immune system dysfunction, encompassing 2 main types: Crohn’s disease (CD) and ulcerative colitis (UC). In CD, inflammation can affect any part of the gastrointestinal tract, from the mouth to the anus, often appearing in discontinuous segments of the small and large intestines. In contrast, UC primarily impacts the colon, with inflammation occurring continuously in the distal regions of the bowel.^1^ Despite advancements in treatment, IBD remains a significant global health challenge, having a profound impact on patients’ quality of life, and its prevalence is notably rising in industrialized countries.^2^^,^^3^ Recent studies have identified genetic and environmental factors, such as lifestyle, diet, antibiotic use, and smoking, as significant contributors to the development of IBD.^4^^,5^ Furthermore, other autoimmune diseases may coexist with IBD, potentially increasing the risk of its occurrence.^6^

Autoimmune skin diseases, particularly vitiligo, are among the common disorders that not only affect skin health but can also be associated with other systemic conditions. Vitiligo is a chronic disease characterized by the loss of skin pigmentation, manifesting in specific areas due to an immune system attack on melanocytes.^7^ This condition primarily presents as white patches without other symptoms such as itching or pain, and it can occur on any part of the skin, especially in areas exposed to sunlight, such as the face and hands.^8^ Vitiligo has a global prevalence of 0.5%-2% and can affect individuals of all ages and ethnic backgrounds, though it is more commonly observed in young people and during adolescence.^9^ While vitiligo is largely considered a cosmetic concern, growing evidence suggests that this disorder may be linked to other autoimmune diseases, including IBD, indicating its potential association with internal systemic conditions.^10^

Recent epidemiological and clinical evidence suggests a significant association between vitiligo and the onset of IBD.^11^ This association is likely reflective of shared immunogenetic factors and systemic chronic inflammation. In a large population study conducted in South Korea, the risk of developing CD and UC in individuals with vitiligo was found to be 2.14 and 1.89 times higher, respectively, compared to the general population.^12^ Additionally, a recent study by Schonmann et al^13^ showed that less than 0.5% of vitiligo patients were diagnosed with UC or CD, yet the prevalence of IBD was higher in comparison to those without vitiligo. Collectively, these findings emphasize the need for targeted and more in-depth exploration of this clinical association.

Given the scattered evidence regarding the connection between vitiligo and IBD, conducting a systematic review aimed at more accurately assessing the prevalence of IBD in these patients could fill the existing gap in the literature. Therefore, the authors set out to explore the prevalence of IBD in individuals with vitiligo through a systematic review and meta-analysis. This study, by synthesizing and analyzing these structured pieces of evidence, will enable the identification of epidemiological patterns and could provide a foundation for the development of targeted screening.

## Materials and Methods

### Study Design

The present study is a systematic review and meta-analysis. The report of this study follows the Preferred Reporting Items for Systematic Reviews and Meta-Analyses (PRISMA) checklist guidelines.^14^

### Search Strategy

In this article, 2 researchers independently searched the Scopus, PubMed/Medline, Web of Science, and Embase databases using both MeSH and non-MeSH keywords, including Inflammatory Bowel Diseases, Crohn Disease, Ulcerative Colitis, IBD, vitiligo, prevalence, and Epidemiology up until March 31, 2025 (Supplementary Table 1). Additionally, gray literature was examined using the Google Scholar database. A reference search was also conducted for related articles to explore further. Finally, using EndNote X7 software (Clarivate Analytics; Philadelphia, USA), all the relevant articles were collected, and duplicates were removed using the same software.

### Eligibility Criteria

All observational (cross-sectional) studies that examined the co-occurrence of IBD or any of its subtypes (UC or CD) in patients with vitiligo, regardless of time or location, were included in this study. These studies had to report the precise prevalence of IBD or one of its subtypes in patients who were solely diagnosed with vitiligo. Studies such as reviews, case reports, case series, meta-analyses, as well as in vitro and in vivo studies were excluded from the analysis. Additionally, studies that did not report the exact prevalence of IBD or any of its subtypes, or those lacking sufficient data to accurately determine the prevalence of IBD, UC, or CD, were excluded. Studies focusing on the prevalence of vitiligo in patients with IBD were also not included. Furthermore, studies with duplicate data were excluded from the review.

In this study, IBD was categorized based on its subtypes into 3 groups: IBD (overall), UC, and CD.^15^ Additionally, the income index of countries was obtained using data from the World Bank Group and the United Nations Development Program.^16^^,^^17^

### Quality Assessment

The risk of bias in the included studies was assessed using the Joanna Briggs Institute critical appraisal tools.^18^ The studies were then categorized as having low, moderate, or high risk of bias.

### Screening of Studies and Data Extraction Form

In this review, 2 researchers independently conducted the study screening and data extraction processes. In cases of any discrepancies in the study evaluation, the final decision was made by the third author, who was responsible for the study design. All final articles included in the study process were extracted into a Microsoft Excel 2016 (Microsoft Corporation, Redmond, WA, USA) sheet, which contained data regarding the author’s name, publication year, study duration, mean and SD of patients, gender, country, World Health Organization region, country income level, IBD type, total sample size of vitiligo patients, and the prevalence of IBD. Additionally, studies that separately reported the exact prevalence of IBD or any of its subtypes, as well as investigated vitiligo as a risk factor for the development of IBD by comparing case and control groups based on vitiligo status, had their data extracted individually.

### Statistical Analysis

The data were imported into the STATA 14.0 statistical software (Stata Corporation; TX, USA) for analysis. A random effects model was employed to account for heterogeneity between studies. Heterogeneity was assessed using Cochran’s Q test and Higgins *I*^2^ test,^19^ and the differences between studies were evaluated qualitatively by the researchers.^18^ Forest plots were used to display the effect size of each study and pooled estimates. In the presence of heterogeneity, methods such as meta-regression analysis and subgroup analysis were applied. 

Given the close agreement between the pooled estimates across studies, no variance-stabilizing transformation was applied. Untransformed (raw) proportions were used for the analysis. The pooled odds ratios (ORs) represent crude (unadjusted) values, calculated directly from the raw data of each study. Sensitivity analysis was also conducted to assess the impact of each individual study on the overall outcome. Publication bias was examined using Egger’s test and visually represented through a funnel plot. A *P*-value of less than .05 was considered statistically significant.

## Results

### Study Selection

**Figure 1.**
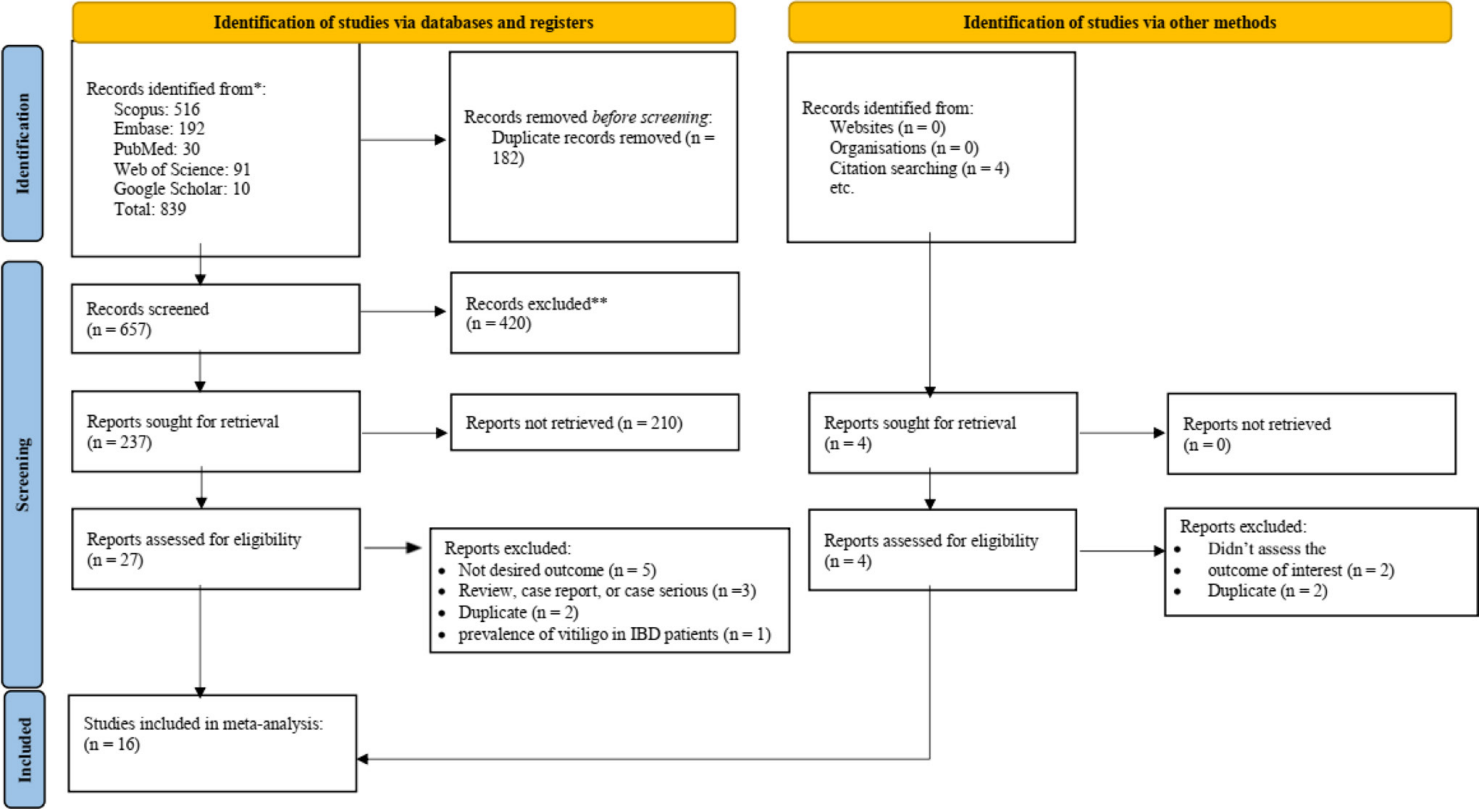
Preferred Reporting Items for Systematic Reviews and Meta-Analyses flow diagram showing the process of study selection, including identification, screening, eligibility assessment, and final inclusion of studies in the meta-analysis.

After searching all national and international databases, a total of 839 articles were identified. After removing duplicates, 657 articles were included for title and abstract screening. Following the review of titles and abstracts, 27 articles advanced to the next stage. In this phase, the full texts of the articles were reviewed, and 16 articles,^11^^-^^13^^,^^20^^-^^32^ consisting of 44 records, were included in the final analysis. The study selection process is presented in Figure 1.

### Study Characteristics

The studies included in the review were published between 2003 and 2024, with data entry spanning from 2000 to 2020. One of the studies^21^ was conducted jointly across 3 countries: the United States, Canada, and the United Kingdom, while the remaining studies were carried out in 4 regions: the Eastern Mediterranean Region (2 studies), the European Region (4 studies), the Region of the Americas (7 studies), and the Western Pacific Region (2 studies). All studies involved both genders, with the average age ranging from 24.5 to 52.3 years in the studies that reported this information. Among these, 1 study specifically reported the prevalence of UC,^20^ and another focused solely on the prevalence of CD.^26^ The quality assessment results of the articles, based on the corresponding checklist, revealed that 9 studies had a low risk of bias, 6 studies had a moderate risk of bias, and 1 study had a high risk of bias. Additional details are provided in Table 1.

### Results of the Meta-Analysis

#### Pooled Prevalence of Inflammatory Bowel Disease and Its Subtypes:

**Figure 2.**
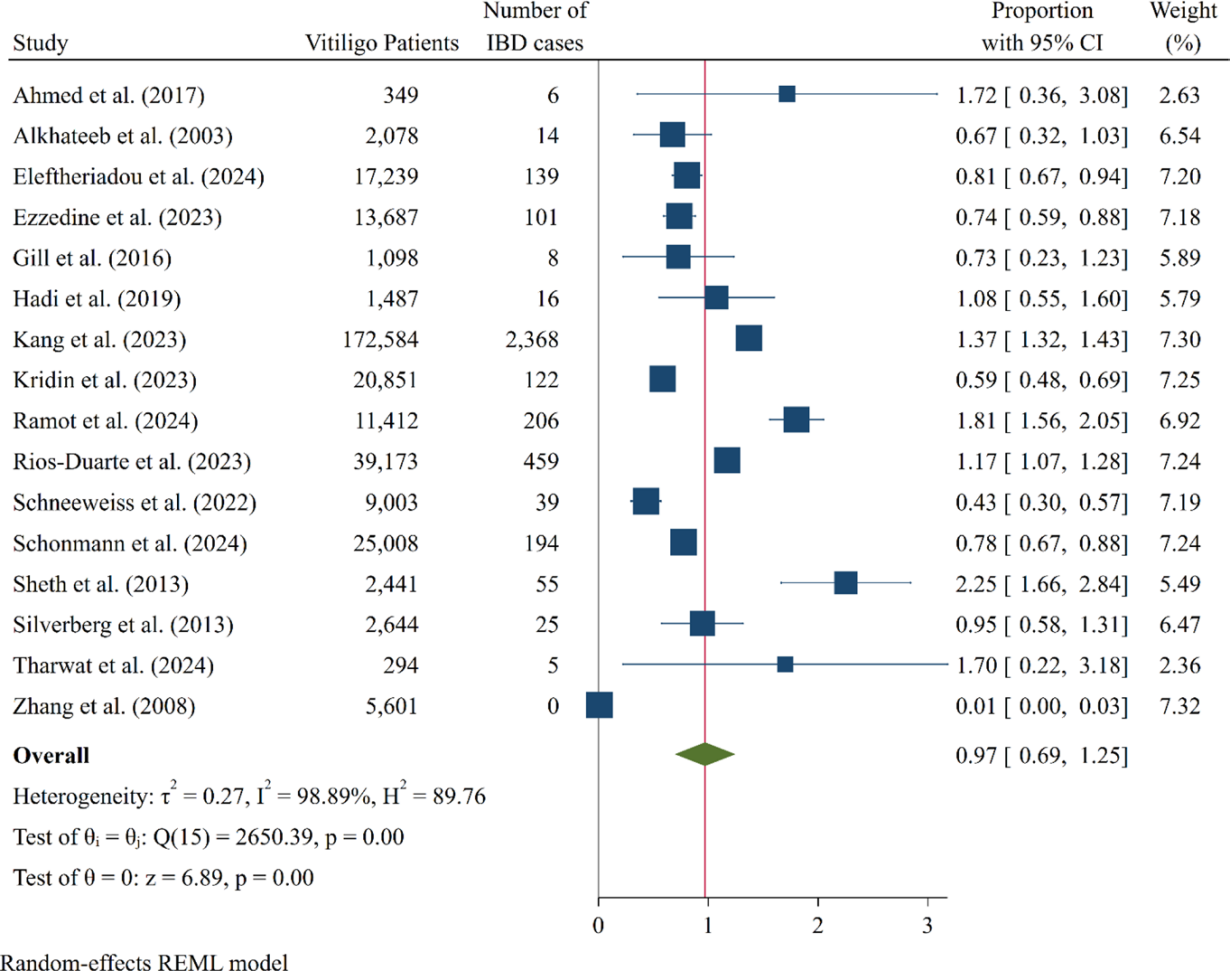
Forest plot depicting the pooled prevalence estimates of inflammatory bowel disease cases among patients with vitiligo, along with 95% CIs for each study.

**Figure 3.**
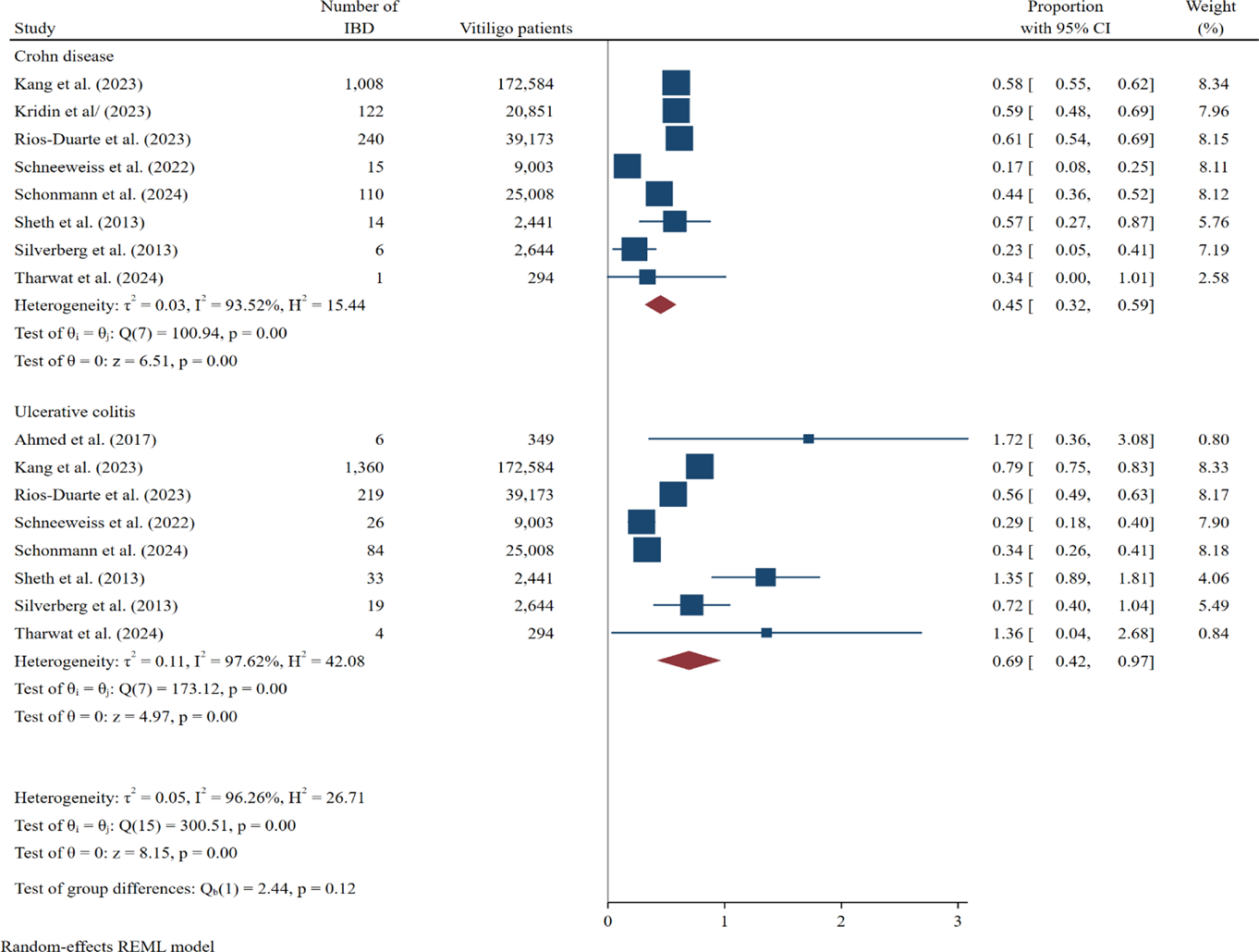
Forest plot depicting the pooled prevalence estimates of Crohn’s disease and ulcerative colitis among patients with vitiligo, along with 95% CIs for each study.

The results of this study indicated that the overall prevalence of IBD in patients with vitiligo was 0.97% (95% CI: 0.69-1.25) (Figure 2). Specifically, the prevalence of CD in these patients was 0.45% (0.32-0.59), while the prevalence of UC was 0.69% (0.42-0.97) (Figure 3). The analysis revealed substantial heterogeneity across studies for IBD (Q = 2650.39, *I*^2^ = 98.89%). In addition, a funnel plot was conducted to assess publication bias in studies on the prevalence of IBD. Furthermore, the Egger test confirmed the presence of this bias (bias: 1.74; *P* = .04) (Supplementary Figure 1).

#### Pooled Prevalence of Inflammatory Bowel Disease Based on Priori-Defined Subgroups:

It was further demonstrated that the pooled prevalence of IBD in studies published before 2020 (0.98%, 95% CI: 0.43-1.53) was similar to that in studies published after 2020 (0.98%, 95% CI: 0.68-1.28). Additionally, studies with a high risk of bias (1.72%, 95% CI: 0.36-3.08) showed a higher pooled prevalence of IBD compared to studies with moderate (1.16%, 95% CI: 0.73-1.59) and low (0.82%, 95% CI: 0.47-1.17) risks of bias. Further details can be found in Table 2.

#### Pooled Prevalence of Inflammatory Bowel Disease Based on Countries and Gender:

**Figure 4.**
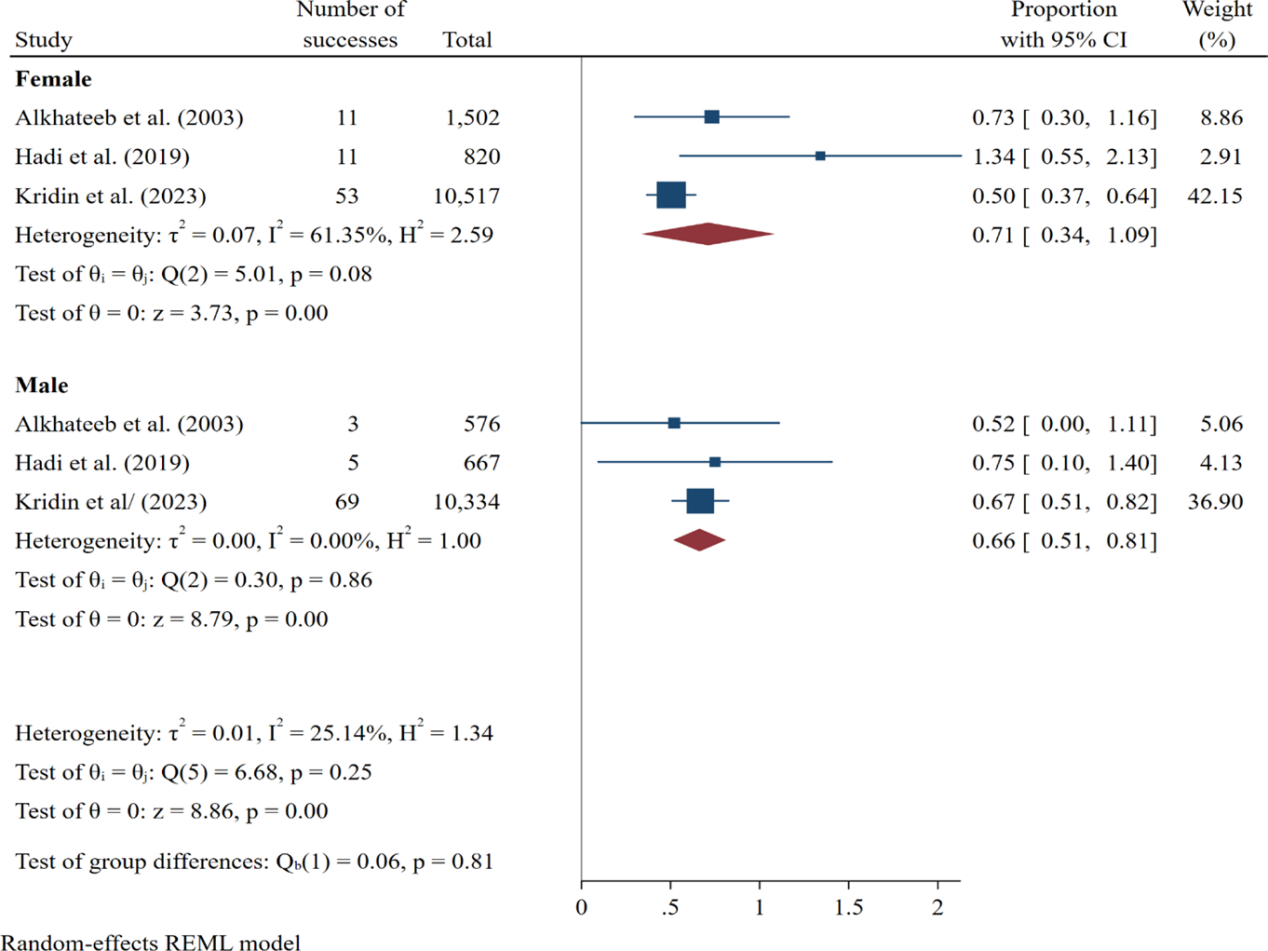
Forest plot illustrating the pooled prevalence of inflammatory bowel disease in vitiligo patients stratified by gender, comparing male and female subgroups.

The pooled prevalence of IBD across different countries ranged from 0.01 to 1.72. The United States, with 7 records, reported a pooled prevalence of IBD at 1.01% (0.62-1.41), while Israel, with 3 records, reported a pooled prevalence of 1.05% (0.31-1.78), ranking second in terms of the number of records. Additionally, the highest pooled prevalence of IBD was reported by Saudi Arabia with a single record, at 1.72% (0.36-3.08). Further subgroup analysis based on study design showed that retrospective cohort studies reported a slightly higher pooled prevalence (1.03%; 95% CI: 0.69-1.37) compared to cross-sectional studies (0.86%; 95% CI: 0.24-1.48). Similarly, studies using clinical examination as the diagnostic method reported consistent estimates (0.97%; 95% CI: 0.67-1.27), while questionnaire-based studies showed wider uncertainty (0.98%; 95% CI: 0.05-1.91) (Table 3). The results (based on 3 records) also showed that the pooled prevalence of IBD was higher in women with vitiligo (0.71%, 95% CI: 0.34-1.09) compared to men (0.66%, 95% CI: 0.51-0.81) (Figure 4).

### Risk Factor Evaluation

**Figure 5.**
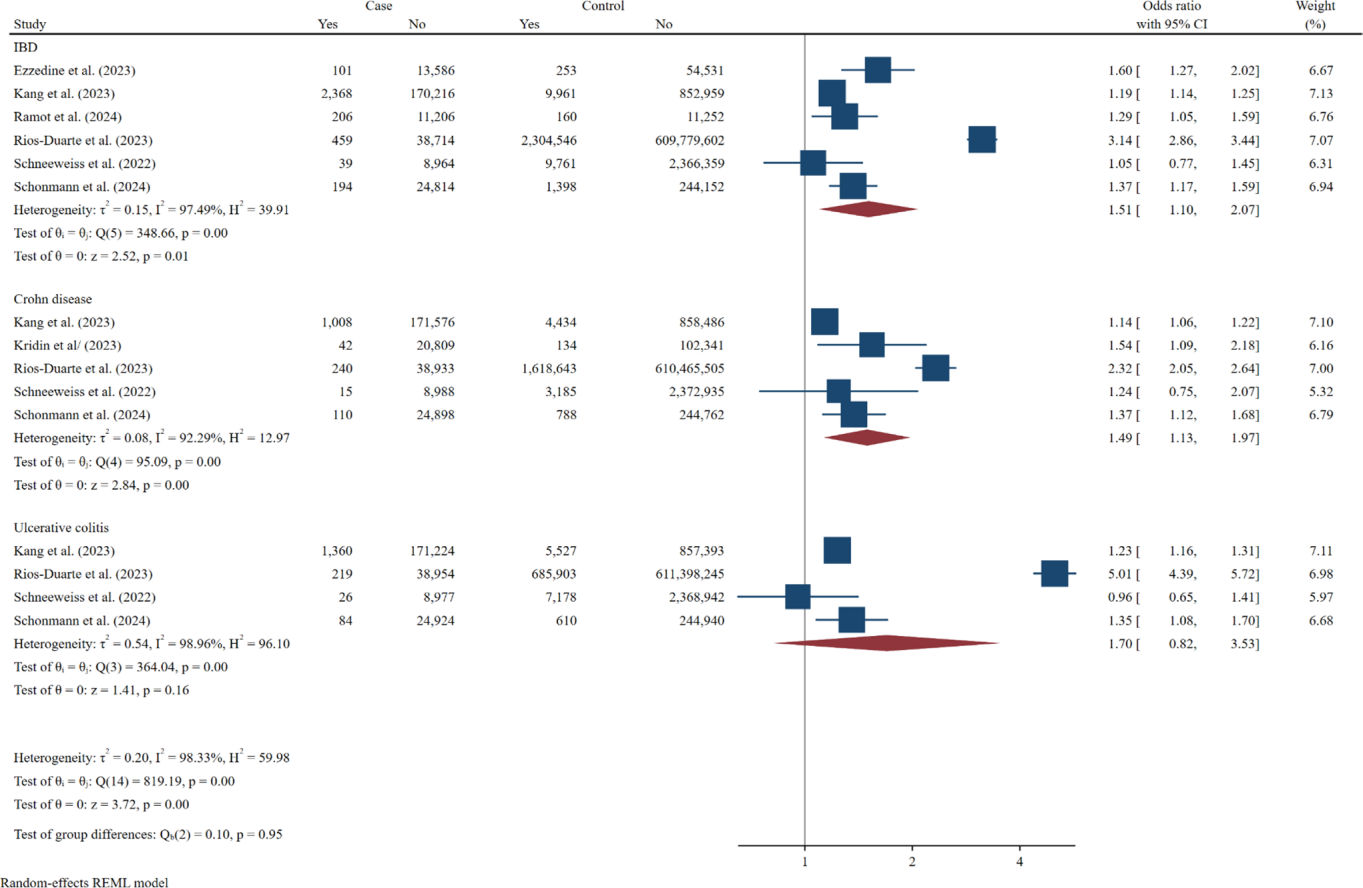
Forest plot of the odds ratios for the association between vitiligo and risk of developing inflammatory bowel disease and its subtypes (Crohn’s disease and ulcerative colitis), based on studies with control groups.

In the analysis of vitiligo as a risk factor for the development of IBD and its subtypes, it was found that among the 6 studies examining vitiligo as a risk factor for IBD, vitiligo was significantly associated with an increased likelihood of developing IBD (OR = 1.51, 95% CI: 1.10-2.07). Furthermore, among 5 studies focused on CD, it was shown that vitiligo significantly increases the prevalence of CD (OR = 1.49, 95% CI: 1.13-1.97). However, in the 4 studies related to UC, vitiligo was not identified as a significant risk factor for the onset of UC (OR = 1.70, 95% CI: 0.82-3.53) (Figure 5).

### Meta-Regression of Prevalence of Inflammatory Bowel Disease Based On

**Figure 6.**
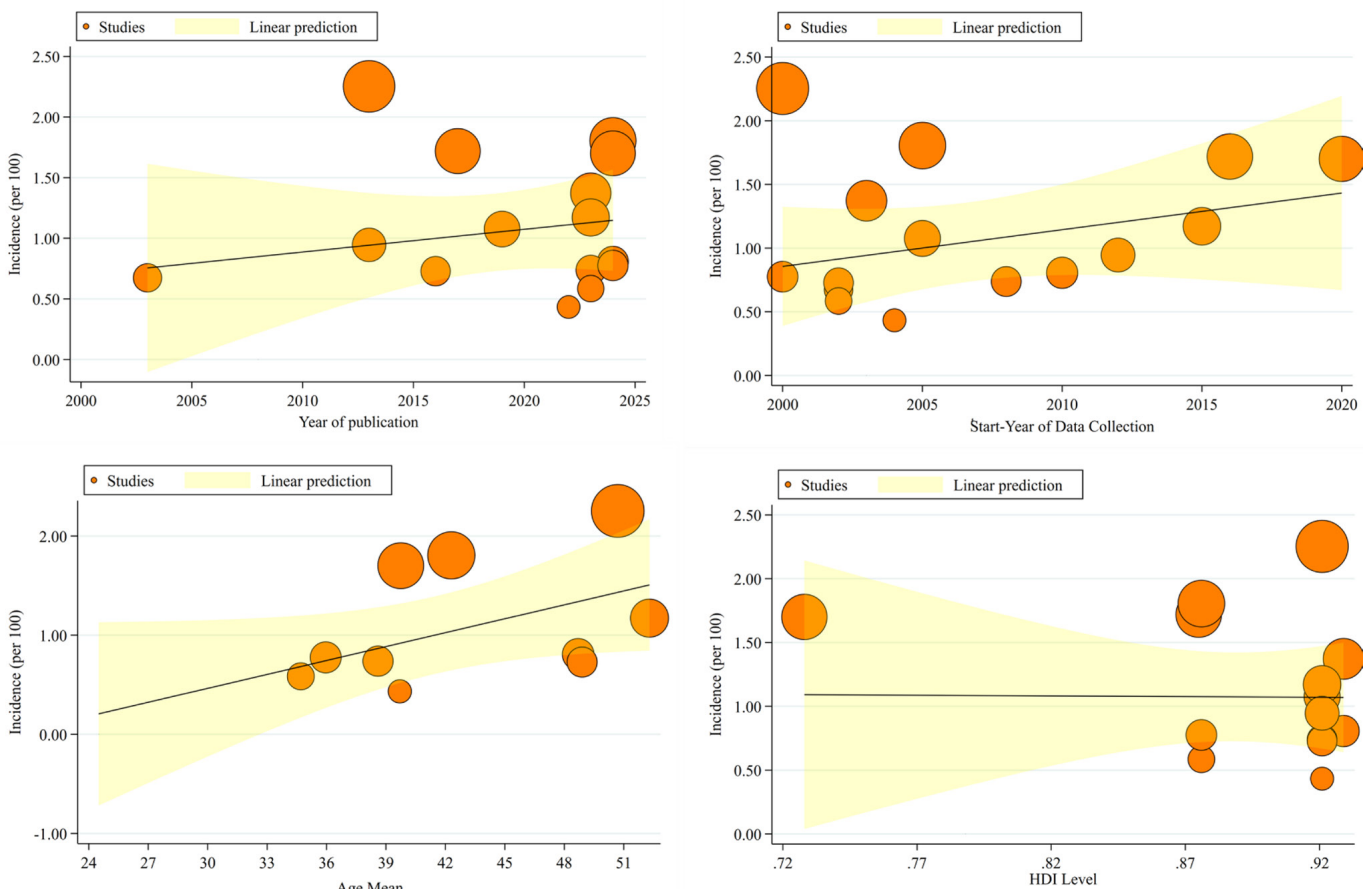
Meta-regression plots analyzing the association between the pooled prevalence of inflammatory bowel disease and 4 moderator variables: (A) Publication year, (B) start year of data collection, (C) mean age of patients, and (D) Human Development Index level of the study country.

According to the analysis of studies based on publication years, it was observed that as the years approach 2025, the pooled prevalence of IBD increases, but this change is not statistically significant (Reg Coef = 0.019, 95% CI: −0.03 to 0.70, *P* = .443). Additionally, when examining studies by data entry years, the pooled prevalence of IBD also shows a nonsignificant increase as the years progress from 2000 to 2020 (Reg Coef = 0.029, 95% CI: −0.02 to 0.80, *P* = .257). Furthermore, when considering the studies based on the average age of patients, it was found that as the average age increases from 24 years to 51 years, the pooled prevalence of IBD rises, though this increase is not statistically significant (Reg Coef = 0.047, 95% CI: −0.01 to 0.91, *P* = .06). Lastly, when analyzing studies based on Human Development Index (HDI), no significant change was observed in the pooled prevalence of IBD as the HDI level increased (Reg Coef = −0.106, 95% CI: −6.30 to 6.09, *P* = .971) (Figure 6).

### Sensitivity Analysis

The sensitivity analysis results indicated that excluding any individual study did not cause a significant change in the effect size, and the findings remained statistically significant (Supplementary Figure 2).

## Discussion

Our study found that the prevalence of IBD in patients with vitiligo was approximately 1%, with slightly lower rates observed for specific subtypes such as UC and CD. Furthermore, the prevalence of IBD in countries such as the United States (from the Region of the Americas) and Israel (from the European Region), where more than 1 record was available, was close to 1%. These findings align with the results of similar studies. For example, a study by Alkhateeb et al^21^ in high-income countries (the United States, the United Kingdom, and Canada) found that the prevalence of IBD among 2078 patients with vitiligo was 0.67%. Another study by Gill et al^24^ in the United States reported a prevalence of 0.73% in vitiligo patients. Similarly, a study by Eleftheriadou et al^22^ in the UK also showed that the prevalence of IBD in patients with vitiligo was less than 1%.^22^ Additionally, a study by Ezzedine et al^23^ in the US and Kridin et al^26^ in Israel confirmed that the prevalence of IBD in vitiligo patients was under 1% (24, 27). However, a study by Ramot et al^11^ in Israel, involving 11 412 vitiligo patients, reported a prevalence of 1.81%. Other studies, such as those by Sheth et al^29^ in the US and Tharwat et al^31^ in Egypt, reported prevalences of IBD greater than 1%. Overall, various studies generally agree that the prevalence of IBD in patients with vitiligo is typically around 1%, though some studies report higher or lower rates. This variability can be attributed to multiple factors, including geographic differences and healthcare access. For instance, the studies conducted in regions with better healthcare access (e.g., the US, UK, and Israel) may be more likely to diagnose IBD in early or milder cases, leading to higher reported prevalence rates. In contrast, regions with limited healthcare access may underreport cases, resulting in lower estimates. Additionally, methodological differences, such as the use of clinical examination vs. questionnaire-based diagnoses, may introduce variability in prevalence measurements. Also, an analysis of study risk of bias revealed that studies with low risk of bias tended to report slightly lower prevalence rates, whereas studies with higher risk of bias reported higher prevalence estimates. This pattern suggests that methodological quality plays a crucial role in the findings, and caution should be exercised when interpreting the data from studies with higher bias risk. However, it is important to note that the prevalence findings for certain countries, such as Saudi Arabia, are based on very limited data, with the highest prevalence reported from a single record. Furthermore, the geographic distribution of the studies included in this analysis poses limitations in generalizing the findings across different populations. Future research should aim to address these gaps by incorporating more data from diverse geographic regions and considering additional factors that may contribute to the observed associations.

Various studies generally indicate that the prevalence of IBD in patients with vitiligo is typically less than 1%, particularly for the CD subtype, which has consistently been reported as less than 1% in nearly all studies.^12^^,^^26^^,^^27^ Regarding UC, except for 3 studies that reported a prevalence greater than 1%,^20^^,^^29^^,^^31^ most studies have shown that the prevalence of UC in vitiligo patients is generally less than 1%. For example, a study by Schneeweiss et al^28^ reported a prevalence of only 0.17% for UC among vitiligo patients. However, this study revealed that the prevalence of UC in vitiligo patients was slightly higher than that of CD. This difference may be attributed to the pathophysiological distinctions between UC and CD; while UC is primarily confined to the colon, CD can affect any part of the gastrointestinal tract.^33^^,^^34^ Additionally, various hypotheses, including genetic, immune, and environmental factors, may contribute to the increased prevalence of UC in patients with vitiligo.^35^^,^^36^ Overall, these findings are likely due to pathological similarities between these diseases, resulting from comparable immune dysfunctions. Therefore, based on the existing research, UC is generally observed in less than 1% of vitiligo patients, but the reported differences may be due to factors such as research methodologies and target populations.

The results of this study showed that the prevalence of IBD based on gender was slightly higher in women than in men. Specifically, a study by Alkhateeb et al^21^ found that 0.73% of the female vitiligo population developed IBD, compared to 0.52% of the male population. Additionally, in a study by Hadi et al,^25^ women had double the prevalence of men. However, in a study by Kridin et al,^26^ focusing on one of the IBD subtypes, men had a higher prevalence of 0.67%, compared to 0.50% in women. These differences in IBD prevalence between genders may be attributed to several factors, including hormonal and immune system differences. Numerous studies have shown that hormonal factors, particularly estrogen, can play a significant role in the development of autoimmune diseases and may especially increase inflammatory responses in women.^37^^,^^38^ Moreover, some studies suggest that due to differences in healthcare access and disease diagnosis, women in certain populations may be more likely to receive an IBD diagnosis, potentially contributing to the higher prevalence in females.^39^ Consequently, based on these findings and existing research, it appears that the prevalence of IBD may be higher in women than in men in certain cases, which could be related to genetic, hormonal, and social differences. However, it is important to note that the results related to gender should be interpreted with caution, as they are based on only 3 studies. The sample sizes in these studies were relatively small, and more comprehensive research is needed to confirm the observed trends and clarify the underlying factors contributing to gender differences in IBD prevalence.

Ultimately, this study demonstrated that vitiligo may significantly increase the risk of developing IBD and its subtypes, particularly UC, by approximately 1.5 times. Although these findings suggest an increased likelihood of UC in patients with vitiligo, the observed relationship was not statistically significant. These results are consistent with the findings of several previous studies. For instance, a study by Ezzedine et al^23^ reported that 0.7% of patients with vitiligo developed IBD compared to 0.5% in the control group, and this study found a significant association between vitiligo and the prevalence of IBD and its subtypes. Similarly, a study by Kang et al^12^ showed that IBD subtypes were significantly associated with an increased risk of CD (16%) and UC (21%).^12^ However, in this study, UC was specifically associated with a higher prevalence in patients with vitiligo, though this association was not statistically robust. These findings may be attributed to genetic, immune, and environmental differences that contribute to the development of these diseases. Notably, some studies have suggested that vitiligo, as an autoimmune disease, could predispose individuals to IBDs such as IBD. In this context, shared genetic factors and similar immune responses may play a critical role in the development of both diseases. These pathophysiological overlaps could explain the increased risk of IBD in patients with vitiligo.^12^ Furthermore, hormonal, genetic, and environmental differences may also influence the co-occurrence of these diseases in vitiligo patients. 

When interpreting the results of this study, it is important to consider its limitations. The first limitation is the limited number of studies that have specifically examined the prevalence of IBD in patients with vitiligo. This scarcity of studies may affect the generalizability of the findings. Additionally, it is important to interpret the pooled prevalence estimate with caution due to the substantial heterogeneity observed across studies. This variability reflects differences in study design, sample size, geographic region, and diagnostic methods. While subgroup and sensitivity analyses support the robustness of the overall findings, the summary estimate should not be viewed as universally generalizable but rather as a descriptive aggregation of heterogeneous data. Moreover, the inconsistency in the diagnostic status of patients is another limitation, as some studies assessed the status of vitiligo patients using questionnaires and self-reporting, which may not provide accurate enough data and could lead to incomplete or imprecise information for the researchers. Another important limitation is the possibility of publication bias. Studies that found low or nonsignificant associations between vitiligo and IBD may be less likely to have been published, leading to their exclusion from the evidence base. This phenomenon, known as selective publication, can artificially inflate the pooled prevalence estimate by over-representing studies with stronger or statistically significant results. The asymmetry observed in the funnel plot (Supplementary Figure 1) supports this concern, indicating that the true prevalence may be lower than the pooled estimate suggests. Readers should interpret the summary findings with this potential distortion in mind. Another limitation of this meta-analysis is the lack of information on potential confounders such as smoking status, biologic therapy, vitiligo severity, and healthcare utilization in many of the included datasets. These factors could contribute to part of the observed association between vitiligo and IBD. The absence of such data means that the potential influence of these variables on the findings could not be assessed in the analysis. Future studies should aim to account for these confounders to better clarify the nature of the association. Finally, the lack of detailed baseline information, such as the stage of vitiligo, its distribution, and treatment regimen, could serve as a confounding factor in the study results, as this information may significantly impact the interpretation and analysis of the data.

This study found that the co-occurrence of IBD in patients with vitiligo is approximately 0.97%. Furthermore, no significant differences in the prevalence of IBD and its subtypes (CD and UC) were observed across various studies and geographic regions. The research also revealed that vitiligo significantly increases the risk of developing IBD and its subtypes. Healthcare providers, particularly gastroenterologists and dermatologists, should be aware of this association and consider IBD as a differential diagnosis in vitiligo patients who present with gastrointestinal symptoms. Additionally, preventing and early identification of IBD risk factors in these patients is crucial. Healthcare systems should focus on educating vitiligo patients about the importance of regular check-ups to ensure any underlying risk factors for IBD are promptly identified and managed.

## Supplementary Materials

Supplementary Material

## Figures and Tables

**Table 1.** Characteristics of Included Studies Reporting Prevalence of Inflammatory Bowel Disease in Vitiligo Patients

Study (REF)	Data Collection Period	Study Design	Country	Age range (Mean ± SD)	IBD Type	Vitiligo Patients (n)	IBD Cases (n)	Risk of Bias
Ahmed et al (2017)^20^	2016-2017	CS	Saudi Arabia	NA	UC	349	6	High
Alkhateeb et al (2003)^21^	2002-2003	CS	USA/Canada/UK	1-95 (NA)	IBD	2078	14	Moderate
Eleftheriadou et al (2024)^22^	2010-2021	RC	UK	12-99	IBD	17 239	139	Low
Ezzedine et al (2023)^23^	2008-2020	RC	USA	12-64 (38.60 ± 14.0)	IBD	13 687	101	Low
Gill et al (2016)^24^	2002-2012	RC	USA	4-100 (48.90 ± 14.60)	IBD	1098	8	Moderate
Hadi et al (2019)^25^	2005-2015	RC	USA	NA	IBD	1487	16	Moderate
Kang et al (2023)^12^	2003-2019	RC	South Korea	NA	IBD	172 584	2368	Moderate
Kridin et al (2023)^26^	2002-2019	RC	Israel	0-96 (34.70 ± 22.40)	CD	20 851	122	Low
Ramot et al (2024)^11^	2005-2021	RC	Israel	NA (42.30 ± 21.30)	IBD	11 412	206	Low
Rios‑Duarte et al (2023)^27^	2015-2019	CS	USA	NA (52.30 ± 0.46)	IBD	39 173	459	Low
Schneeweiss et al (2022)^28^	2004-2020	RC	USA	NA (39.70 ± 21.20)	IBD	9003	39	Low
Schonmann et al (2024)^13^	2000-2023	RC	Israel	NA (35.96 ± 22.39)	IBD	25 008	194	Low
Sheth et al (2013)^29^	2000-2011	RC	USA	NA (50.70)	IBD	2441	55	Moderate
Silverberg et al (2013)^30^	2012-2013	PC	USA	NA	IBD	2644	25	Moderate
Tharwat et al (2024)^31^	2020-2020	CS	Egypt	3-86 (39.75 ± 14.52)	IBD	294	5	Low
Zhang et al (2008)^32^	2003-2007	CS	China	1-91 (24.50 ± 14.60)	IBD	5601	0	Low

CD, Crohn’s disease; CS, cross-sectional; IBD, inflammatory bowel disease; NA, not mentoned; RC, retrospective cohort; UC, ulcerative colitis.

**Table 2.** **C**ountry-Wise Pooled Prevalence Estimates of Inflammatory Bowel Disease Among Patients with Vitiligo

Country*	No. of Datasets	Vitiligo Patients (n)	IBD Cases (n)	Pooled Prevalence % (95% CI)	Weight (%)	Heterogeneity	
						Q	*I*^2^ (%)
USA	7	69 533	703	1.01 (0.62-1.41)		96.12	95.88
Israel	3	57 271	522	1.05 (0.31-1.78)		81.24	98.89
China	1	5601	0	0.01 (0.0-0.03)		–	–
Egypt	1	294	5	1.70 (0.22-3.18)		–	–
South Korea	1	172 584	2368	1.37 (1.32-1.43)		–	–
Saudi Arabia	1	349	6	1.72 (0.36-3.08)		–	–
United Kingdom	1	17 239	139	0.81 (0.67-0.94)		–	–

IBD, inflammatory bowel disease.

*Countries are sorted based on the number of datasets.

**Study of Alkhateeb et al^21^ (2003) was not included due to its multicenter data collection method.

**Table 3.** Subgroup Analysis of Pooled Prevalence of Inflammatory Bowel Disease. in Vitiligo Patients Based on Study and Population Characteristics

Variable	No. of Datasets	Vitiligo Patients (n)	IBD Cases (n)	Pooled Prevalence % (95% CI)	Heterogeneity	
					Q	*I*^2^ (%)
Publication year						
Before 2020	7	15 698	124	0.98 (0.43-1.53)	122.51	93.96
2020-2025	9	309 251	3633	0.98 (0.68-1.28)	387.08	98.20
Sampling start year						
Before 2005	8	238 664	2800	0.82 (0.38-1.27)	2151.25	99.50
2005-2025	8	86 285	957	1.13 (0.83-1.44)	74.18	92.34
Risk of bias						
High	1	349	6	1.72 (0.36-3.08)	0	–
Moderate	6	182 332	2486	1.16 (0.73-1.59)	35.35	90.24
Low	9	142 268	1265	0.82 (0.47-1.17)	1020.75	99.0
Country income level						
Middle	2	5895	5	0.69 (0.0-2.31)	5.03	80.13
High	13	316 976	3738	1.05 (0.78-1.32)	2647.16	97.71
Type of Study						
Cross-sectional	5	47 495	484	0.86 (0.24-1.48)	455.60	98.54
Retrospective cohort	10	274 810	3248	1.03 (0.69-1.37)	398.68	98.35
Prospective cohort	1	2644	25	0.95 (0.58-1.31)	–	–
IBD diagnisis method						
Questionnaire	2	2427	20	0.98 (0.05-1.91)	2.12	52.76
Clinical examination	14	322 522	3737	0.97 (0.67-1.27)	2643.29	99.09

IBD, inflammatory bowel disease.

## Data Availability

The study included all data in the main manuscript and supplementary files. Further data that support the findings of this study are available from the corresponding authors upon reasonable request.
