# Supplementary material for: Co-Occurrence of Inflammatory Bowel Disease in Patients with Vitiligo: A Systematic Review and Meta-Analysis
Source: Turk J Gastroenterol. 2026 Jan 2;37(1):3–14. doi: 10.5152/tjg.2026.25282 (PMC12824891; doi:10.5152/tjg.2026.25282)
Supplement: Supplementary Material [file supplementary_material.pdf]

Supplementary Table 1. Search strategy

PubMed (ALL Fields)	(((“Prevalence”[Mesh] OR “Epidemiology”[Mesh] OR “epidemiology” [Subheading]) OR “Incidence”[Mesh]) AND (((((((“Inflammatory Bowel Diseases”[Mesh]) OR “Crohn Disease”[Mesh]) OR “Colitis, Ulcerative”[Mesh]) OR “Colitis”[Mesh]) OR “Enteritis”[Mesh]) OR (IBD)) OR (UC)) OR (CD))) AND ((((“Vitiligo”[Mesh]) OR (pigmentary)) OR (leucoderma)) OR (leukoderma))
Scopus (TITLE-ABS-KEY)	TITLE-ABS-KEY ((“Prevalence” OR “Incidence” OR “Epidemiology” OR “Prevalences” OR “Epidemiologies” OR “Epidemics” OR “Morbidity” OR “Outbreaks” OR “Surveillance” OR “Endemics” OR “Occurrence” OR “Frequency” OR “Incidences” OR “Attack Rate” OR “Attack Rates” OR “Rate, Attack” OR “Person-time Rate” OR “Person time Rate” OR “Person-time Rates” OR “Rate, Person-time” OR “Rate, Secondary Attack”) AND (“Inflammatory Bowel Diseases” OR “Inflammatory Bowel Disease” OR “Bowel Diseases, Inflammatory” OR “Crohn’s Disease” OR “Crohns Disease” OR “Crohn’s Enteritis” OR “Enteritis” OR “Crohn Disease” OR “Colitis” OR “Ileocolitis” OR “Ileitides” OR “Ileitis” OR “Idiopathic Proctocolitis” OR “Ulcerative Colitis” OR “Enteritides” OR “IBD” OR “UC” OR “CD”) AND (“vitiligo” OR “leukoderma” OR “pigmentary” OR “leucoderma”))

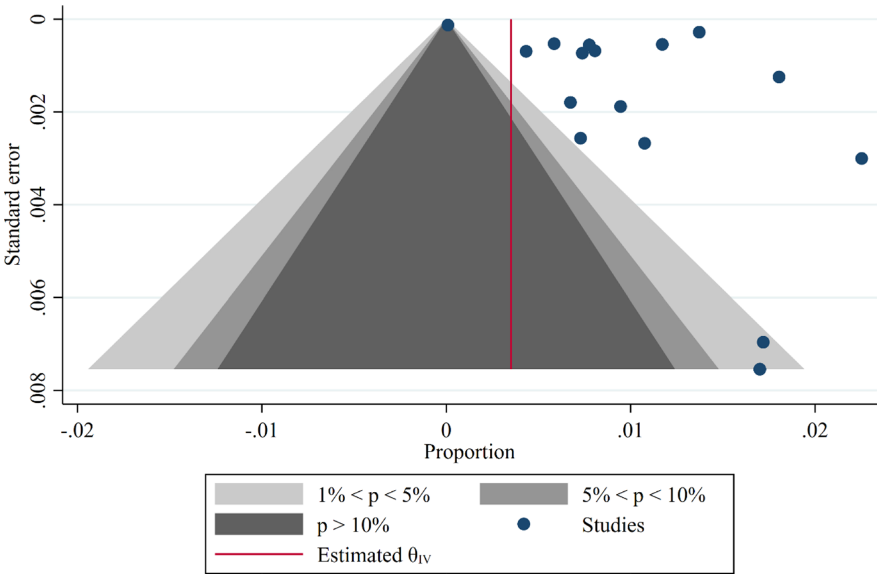

Supplementary Figure 1. Funnel plot of standard error by proportions for the assessment of publication bias.

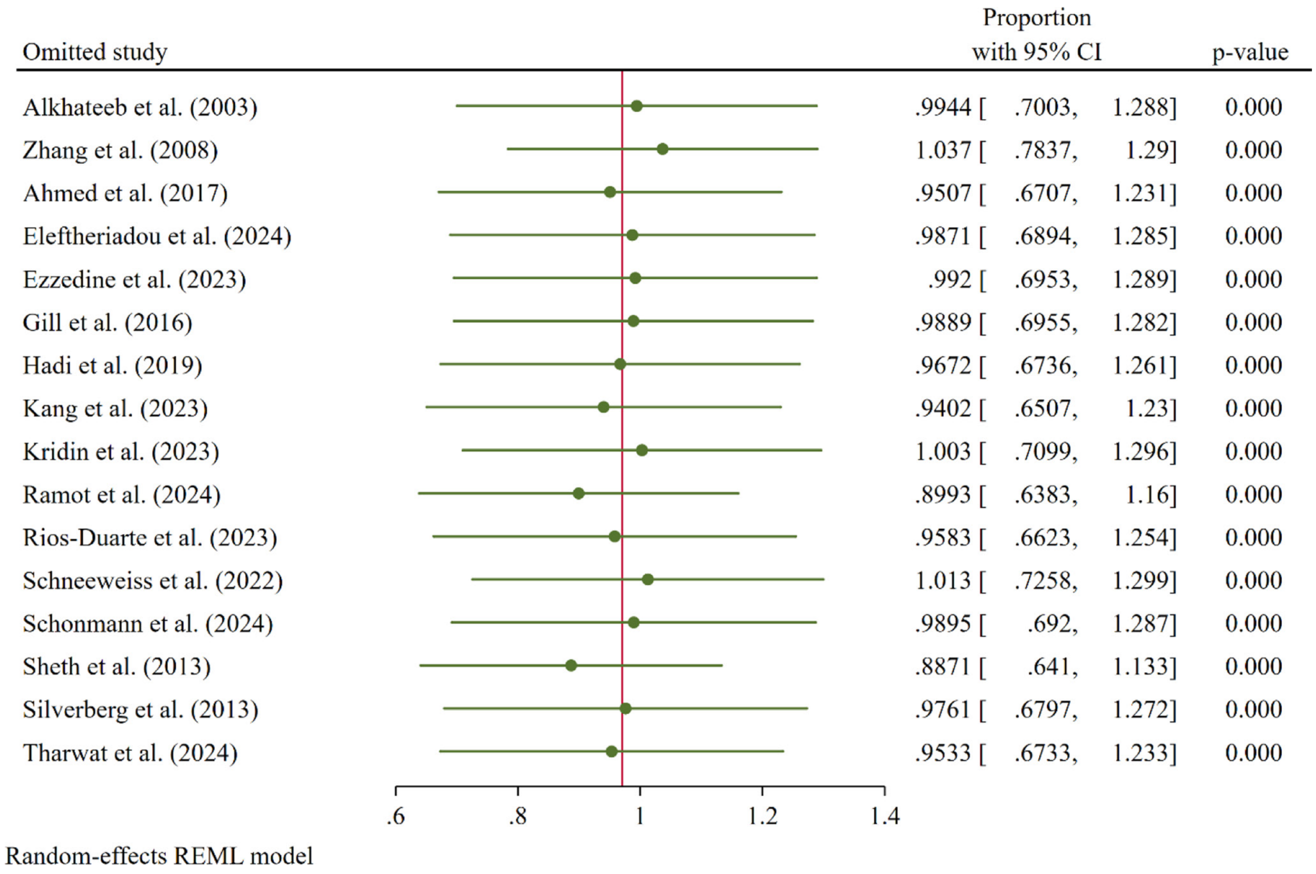

Supplementary Figure 2. Sensitivity analyses of included studies.
